# How space-number associations may be created in preliterate children: six distinct mechanisms

**DOI:** 10.3389/fpsyg.2015.00215

**Published:** 2015-03-05

**Authors:** Hans-Christoph Nuerk, Katarzyna Patro, Ulrike Cress, Ulrike Schild, Claudia K. Friedrich, Silke M. Göbel

**Affiliations:** ^1^Department of Psychology, Eberhard Karls University of TuebingenTuebingen, Germany; ^2^Leibniz Institute for Knowledge Media, Knowledge Media Research Center, TuebingenGermany; ^3^LEAD Graduate School, Eberhard Karls University of TuebingenTuebingen ,Germany; ^4^Department of Psychology, University of WarsawWarsaw, Poland; ^5^Department of Psychology, University of YorkYork, UK

**Keywords:** space-number associations, reading acquisition, numerical development, literacy, preliteracy, SNARC, number acquisition

## Abstract

The directionality of space-number association (SNA) is shaped by cultural experiences. It usually follows the culturally dominant reading direction. Smaller numbers are generally associated with the starting side for reading (left side in Western cultures), while larger numbers are associated with the right endpoint side. However, SNAs consistent with cultural reading directions are present before children can actually read and write. Therefore, these SNAs cannot only be shaped by the direction of children’s own reading/writing behavior. We propose six distinct processes – one biological and five cultural/educational – underlying directional SNAs before formal reading acquisition: (i) *Brain lateralization,* (ii) *Monitoring adult reading behavior,* (iii) *Pretend reading and writing, and rudimentary reading and writing skills,* (iv) *Dominant attentional directional preferences in a society, not directly related to reading direction,* (v) *Direct spatial-numerical learning,* (vi) *Other spatial-directional processes independent of reading direction.* In this mini-review, we will differentiate between these processes, elaborate when in development they might emerge, discuss how they may create the SNAs observed in preliterate children and propose how they can be studied in the future.

## THE READING AND WRITING DIRECTION ACCOUNT IN ADULTS

One of the most intriguing findings in the field of Numerical Cognition is that numbers in adults are automatically associated with a spatial horizontal dimension (Fischer and Shaki,2014). In Western countries, relatively larger numbers are usually associated with the right side in space and smaller numbers with the left side in space. The most widely studied demonstration of such an association is the so-called SNARC-effect (Spatial-Numerical Association of Response Codes; Dehaene et al., 1993): even in tasks in which number magnitude is irrelevant (e.g., parity judgment tasks), participants are faster to respond to larger numbers with the right hand, and to smaller numbers with the left hand (Wood et al., 2008).

The *common reading account* proposes that the origin of this directionality stems from reading habits. Suggested by Dehaene et al. (1993), this account was further corroborated in a series of studies by [e.g., Shaki and Fischer (2008), Fischer et al. (2009), Shaki et al. (2009); see also Zebian (2005)]. They showed that general and situational exposure to right-to-left writing modulated or even reversed the common SNARC effect – participants exposed to right-to-left reading habits had a null or right-to-left SNARC effect. However, there are other accounts of the origin of SNAs. For instance, some researchers propose that the SNARC effect is created by the order of numbers in verbal working memory sequences (e.g., van Dijck and Fias, 2011). Others suggest that the direction of the SNARC effect might be triggered by early finger counting habits (an embodied account; Fischer, 2008) or that verbal-linguistic markedness might contribute to number-parity and number magnitude representations (Nuerk et al., 2004). A detailed discussion of these accounts is beyond the scope of the current review; here, we will focus on the dominant account, which is the *common reading account*.

## SPACE-NUMBER ASSOCIATIONS IN CHILDREN

Space-number associations (SNAs) develop in early childhood (McCrink and Opfer, 2014). Western preschoolers have a strong preference for left-to-right object counting (Briars and Siegler, 1984; Opfer et al., 2010; Shaki et al., 2012; Knudsen et al., in press) as well as for left-to-right sequences of Arabic digits (Opfer and Furlong, 2011). In a typical counting task, an explicit spatial-numerical decision has to be made, i.e., to start from the left or from the right. However, preschoolers show SNAs even in tasks not requiring an explicit spatial-numerical decision. Patro and Haman (2012) observed a SNARC-like effect in a non-symbolic numerosity comparison task in children as young as 3- and 4-years-old. All these children were clearly preliterate, so their reading habits could not explain their SNAs. In addition, SNAs in preschool children are already automatic and present even when magnitude is not task-relevant. Hoffmann et al. (2013; Experiment 2) observed a classical SNARC effect in children as young as 5;5 years when children had to decide whether Arabic numbers changed to red or to green, by pressing a left- or right-located button. A SNARC-like interaction between number magnitude and response side was observed. Thus, number magnitude was task-irrelevant (children had to decide about color), but automatically activated. Moreover, there was no explicit instruction that magnitude should be related to one side of space. The presence of SNAs in preschool children clearly challenges the common reading account for SNAs, because those children have not yet developed reading habits themselves.

Recently, de Hevia et al. (2014) observed that already 7-months-old infants, growing up in Italy ^1^ (left-to-right-reading), showed a preference for left-to-right increasing sequences of sets’ numerosities. They proposed an alternative to the common reading account and suggested *biological predisposition* to cause SNAs in very young children. These biologically determined SNAs might later be modulated or even reversed by reading/writing acquisition.

Even such a *combination account* of biological left-to-right predisposition and later modulation by cultural reading habits is at odds with recent studies. Shaki et al. (2012) showed that reading/writing habits in a society modulated counting habits already in preliterate children. British 3–6-years-old preschool children counted mainly from left-to-right, whereas the majority of the Israeli and Palestinian children (growing up in right-to-left reading cultures) counted from right-to-left. The combination account cannot explain these data. Its biological component cannot explain any cultural variation by reading habits at all. Its reading experience component cannot explain cultural modulation before reading acquisition.

Spatial-directional training also shapes or modulates SNAs in preliterates. Patro et al. (in press) provided directional attentional non-numerical training to 3–4-years-old children. They observed that left-to-right training led to a subsequent left-to-right SNARC-like effect, while right-to-left training led to a right-to-left SNARC-like effect. In another study, Göbel et al. (2014) tested counting direction in British and Arab preschoolers before and after a 5 min reading-related experience that was either left-to-right or right-to-left. They found that, irrespective of children’s initial counting direction, most children who observed left-to-right reading counted left-to-right, and most children who observed right-to-left reading counted right-to-left. Such modulation of SNA direction by training also speaks against an exclusively biological account.

Both studies clearly show that spatial-directional experience shapes SNAs in preschoolers. In addition, taking both studies together they make an important point, which will drive our review: different SNA types were modulated by different spatial (training) mechanisms. Patro et al. (in press) conducted an implicit attentional training, not related to reading observation, and this training affected an implicit directional measure of SNA (the SNARC effect), but did not lead to a change in explicit counting direction. Similarly, Göbel et al. (2014) showed an effect on explicit counting direction only when the training included explicit reading observation but not with implicit attentional training. This is in line with Kamawar et al.’s (2010) observation that children have a strong idea which explicit SNA is correct. They showed that the majority of 5–11-years-old children they tested in Canada believed that the order in which items were counted was important. Most children favored a left-to-right, top-to-bottom order of counting. Thus, children are very aware of explicit counting direction and have a clear idea of what the ‘correct’ direction of counting is. For children, this ‘correct’ direction seems to be consistent with their particular cultural reading/writing habits.

There is now clear empirical evidence that SNAs can be formed in preschool children, but we still lack a coherent theoretical proposal that could explain which concrete mechanisms or processes contribute to the emergence of number-space effects in young children. This is an obvious gap in this line of research. This mini-review aims to close this gap by proposing and discussing *six distinct mechanisms.*

It is important to note that numbers can be linked to spatial directions in different ways. Patro et al. (2014), who proposed four SNAs in general, described two *spatial-directional* SNA types in particular:

(i) Associations between cardinalities and spatial directions: in this SNA, there is a directional association similar like in a SNARC effect – in left-to-right reading cultures larger numerosities are responded to faster on the right side and smaller numerosities on the left.
(ii) Associations between ordinalities and spatial directions: in this SNA, spatial direction is related to ordinality (e.g., the direction of counting) – it is not necessarily related to cardinality because younger preschoolers do not know that the end point of the counting sequence equals the cardinality (i.e., the total number of objects in the sequence).

The mechanisms outlined in this review may not contribute equally to the emergence of the two SNA types described above. These mechanisms, their differential impact, and the probable age of onset will be defined and systematically demarcated in the remainder of this review.

## MECHANISMS POTENTIALLY INDUCING SPATIAL-NUMERICAL DIRECTIONALITY IN PRELITERATE CHILDREN

### BRAIN LATERALIZATION

Brain lateralization may play an important role for early spatial-directional preferences (Rugani et al., in press, 2015, for animal studies). Directional spatial-numerical biases in 7-months-old infants have been interpreted as an innate disposition to associate larger numerosities with one side in space (de Hevia et al., 2014). While such findings may be explained by innate biases, they are not fully conclusive yet: first, so far, no evidence has been obtained that the spatial-numerical biases vary systematically with an indirect measure of brain lateralization: handedness. Second, early presence of a mechanism does not necessarily imply innateness – 7 months might be long enough to learn about spatial-directional regularities in a social cultural setting. Third, even spatial biases which seem to be strongly predisposed might be subject to cultural influences (Güntürkün, 2003; Shaki, 2013). To be clear, these arguments do not preclude a role of brain lateralization in humans but, in our opinion, the case is far from closed.

### MONITORING ADULT READING BEHAVIOR

Joint book reading activity promotes emergent literacy (including print awareness) in children who are not yet conventional readers (Sénéchal et al., 1996; Mol et al., 2009). Via joint book reading, preliterate children could learn about text directionality by observing their parents pointing to particular places in text or referring to subsequent pictures (Dobel et al., 2007; McCrink et al., 2011). Knowledge of spatial organization of script and pictures in books (and also about the organization of books) could be acquired very early because adults start reading books to children as young as 1–2 years (Sénéchal et al., 1995; Fletcher and Reese, 2005). So, by reading books to children, adults may impose an attentional directionality, which children internalize even before they formally acquire reading skills.

### PRETEND READING AND WRITING, AND RUDIMENTARY READING AND WRITING SKILLS

Children acquire basic aspects of reading and writing well before formal instruction in school starts (Snow et al., 1998). In pretend reading, typically developing children at the end of their third year not only demonstrate that they know how to hold a book and turn pages in their native writing system, but also that they know that stories progress as pages are turned and that a story has a beginning, middle and end (e.g., Doake, 1985; Sulzby, 1985, Valencia and Sulzby, 1991). Also, starting at the end of age 3, approximate word-by-word pointing in pretend reading can be observed (Dooley, 2010). In pretend writing, preliterate children ‘write’ lists, thank-you notes, etc. (Dyson, 1982). Thus, young children at least start extracting the characteristic direction of their native language’s writing system. Between the ages of 3 and 4 children become more and more aware of the elements of writing and their linearity so that most 4 years-old can read and write one or more simple words, including their own name (Hildreth, 1936; Bloodgood, 1999; Puranik et al., 2011, 2013). That is, the directional process related to the local writing system appears to become active at the end of the third year and further elaborated in older preschoolers.

### DOMINANT ATTENTIONAL-DIRECTIONAL PREFERENCES IN A SOCIETY, NOT DIRECTLY RELATED TO READING DIRECTION

Reading and writing habits may influence directional preferences which at first sight have nothing to do with reading and writing themselves. First, visuo-spatial processing appears to be biased by writing direction. For instance, Arabic participants preferred drawing horizontal lines from right-to-left, while English-speaking participants preferred drawing them from left-to-right (Lieblich et al., 1975). Culture-dependent line bisection biases have been observed both in adults (Chokron and Imbert, 1993; Kazandjian et al., 2010; Rinaldi et al., 2014) and preliterate preschoolers (Chokron and De Agostini, 1995; but see Fagard and Dahmen, 2003). Second, spatial imagery also appears to be biased by writing direction. Hindi participants, reading from left to right, drew bicycles or elephants facing to the left, whereas Arab participants exhibited a rightward bias for those objects (Vaid, 1995). For temporal preferences (e.g., meals of the day), adults tended to prefer horizontal alignment corresponding to their reading habits, i.e., future to the right in left-to-right writing systems and future to the left in right-to-left writing systems (Tversky et al., 1991). Furthermore, spatial representations of actions appeared to be modulated by reading direction. Adults exposed to left-to-right writing systems preferentially place and expect agents on the left side of a picture, whereas adults exposed to right-to-left writing systems show the reverse pattern (Maass and Russo, 2003; Dobel et al., 2007; Maass et al., 2009). In sum, adults engage in all kinds of attentional-directional behaviors which are not directly related to reading/writing, but which are nevertheless consistent with the direction of reading/writing in a society. Children may observe such behaviors from parents and other models and imitate them.

Importantly, some culture-dependent spatial directional *actions* themselves do not develop before school: children of school age, but not preschoolers showed culture-dependent directionality in drawing (Kebbe and Vinter, 2013). Similarly, children of school age showed temporal ordering of spatial relations (Tversky et al., 1991), but preschoolers did not show a preference regarding spatial placement of agents (Chokron and De Agostini, 2000; Spalek and Hammad, 2005; Dobel et al., 2007; McCrink et al., 2014; for reviews see Kazandjian and Chokron, 2008; Chokron et al., 2009).

It should be also noted that many applications for electronic devices (computers, tablets, smartphones) are adjusted for different reading/writing directions. Even operating systems (e.g., Windows) have a Hebrew/Arabic version, which starts from right-to-left: the ‘start’ button is located on the right side of the screen and the window menu opens from right-to-left. Similar directional differences can be found in childrens’ applications /games, which are designed for 3–4-years-old kids, who are not yet able to read. Thus, via such applications, young children are directly exposed to certain attentional-directional cultural preferences ^2^.

In sum, there are multiple cultural spatial-directional biases in everyday actions which are not directly related to reading behavior, but are nevertheless consistent with its directionality in the local culture. It is conceivable that such biases influence attentional directionality in preliterate children.

### DIRECT SPATIAL-NUMERICAL LEARNING

The mechanisms described above are concerned with spatial-directional biases which are not related to numbers. However, there are also direct explicit instructions of spatial-numerical relations. For example, children are exposed to certain spatial arrangements of numbers in their picture books, and they are often formally and informally taught to count objects in a certain order. Lindemann et al. (2011) have shown that finger-counting habits also seem to differ between cultures. Finger counting habits even strongly differ between cultures which have the same script [see Bender and Beller, 2012, for between culture-variations; Wasner et al. (in press), for within-culture variations]. Thus, there is a spatial-numerical component in finger counting that goes beyond reading directionality and which is directly learnt in a given culture.

Therefore, children may directly learn certain directionalities of space-number relations from adult models or instruction. This direct instruction of SNAs may begin at about 2–3 years, when children start to count.

### OTHER SPATIAL-DIRECTIONAL PROCESSES INDEPENDENT OF READING DIRECTION

Cultures may also differ in other spatial-directional processes, which are not related to reading direction or explicit numerical instruction. For instance, spatial looking behavior when crossing a street is influenced by the lane on which the traffic usually drives (first look to the right for left-lane traffic in the UK, first look to the left, for right-lane traffic in the rest of Europe). Such spatial-directional mechanisms might affect SNAs as well. However, we are not aware of any studies yet examining such influences. We would hypothesize that other spatial-directional influences generally increase directional SNAs when they are congruent to the cultural reading/writing direction and decrease SNAs when they are incongruent.

## WHERE WE ARE AND WHAT WE CAN CONCLUDE

**FIGURE 1:**
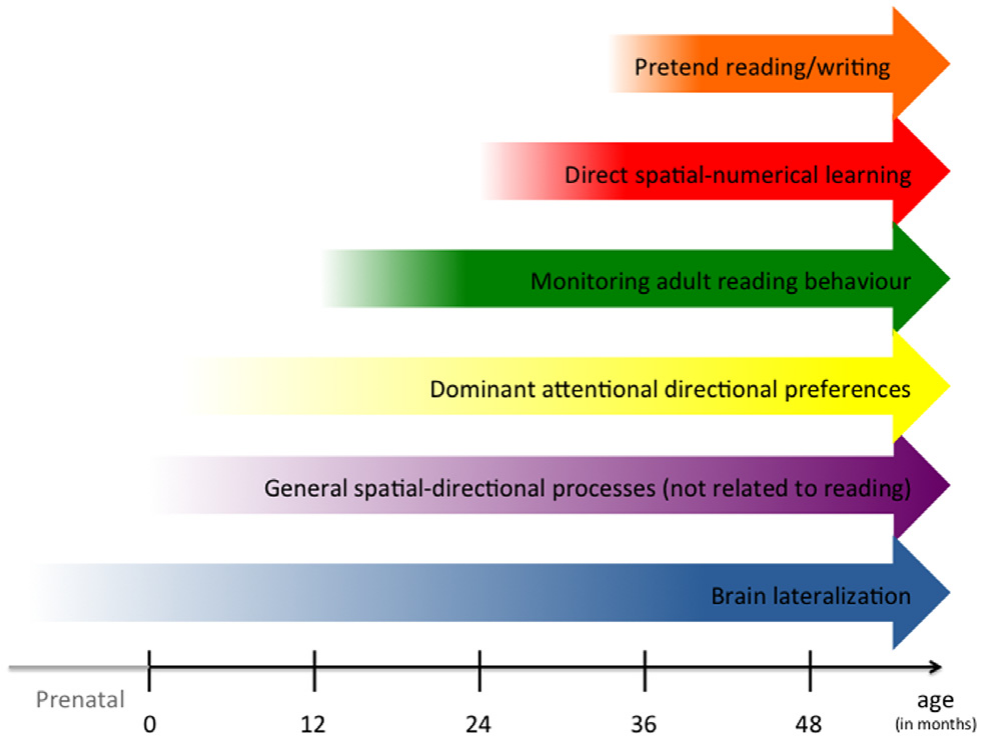
**Overview of the different mechanisms underlying the acquisition of spatial-numerical associations.** Mechanisms are ordered according to their probable age of onset according to the literature. Exact time of onset is often difficult to determine, therefore, the shaded start of the arrows depicts the probable range of onset in typically developing children. Note that brain lateralization starts before birth and that all mechanisms continue to activate spatial-numerical associations beyond the age of 48 months as indicated by the arrows.

We have defined and delineated six distinct mechanisms which might be responsible for the emergence of spatial-numerical directional preferences before formal literacy (for an overview including time of onset, see **Figure 1**). These mechanisms are probably often consistent, but can be sometimes in conflict. For instance, an Arab parent may read Arab children’s books from right-to-left, but may count objects from left-to-right, because this is how numbers are ordered in most numerical and arithmetic graphs. Therefore, different SNA types may be represented in a different fashion or even in an opposite direction because they are learnt by different, possibly directionally conflicting, mechanisms.

Most of the learning mechanisms proposed here are related to embodied spatial-numerical learning (e.g., Fischer and Brugger, 2011; Moeller et al., 2012; Wasner et al., in press). Many spatial-numerical associations are bodily experienced and might be represented in an embodied way, for instance, by using fingers for number magnitude. In recent intervention studies (Fischer et al., 2011; Link et al., 2013, 2014), it was shown that embodied spatial-numerical training leads to greater successful learning than various types of control training. Spatial experiences which are strongly routed in bodily representations may exert stronger influences on the build-up of SNAs, compared to other experiences. A similar account has been proposed by McCrink and Opfer (2014), who suggest that oriented motor behavior (e.g., hand movement during counting) might be a primary factor which refines SNAs in children. Following Fischer and Brugger (2011), one can postulate that for some SNAs embodied cultural influences like dominant reading/writing behavior may be most relevant (ordinality in counting), while for other SNAs (cardinality and its response side association) situated influences are more dominant.

We conclude that spatial-numerical directional preferences before formal reading should not be surprising. They need not be innate, because they may develop through many different cultural and social mechanisms. We suggest that their nature and consistency should be systematically studied. For future studies, we make several predictions:

(i) Explicit SNAs (e.g., counting) should be trained best by explicit spatial-directional experiences, while implicit SNAs (e.g., SNARC) should be learned best through implicit spatial experiences.
(ii) Conflicting spatial directions should lead to weaker directional SNAs than congruent spatial directions.
(iii) Spatial learning mechanisms that are strongly embodied should influence SNAs more than mechanisms that are less strongly embodied or not embodied.

While these predictions are consistent with the available data, they have not been systematically tested so far. Future studies should not focus on the mere existence of different spatial-numerical associations in preschool children, but start exploring the relative contributions of distinct mechanisms which lead to the emergence and shape of distinct SNAs.

## Conflict of Interest Statement

The authors declare that the research was conducted in the absence of any commercial or financial relationships that could be construed as a potential conflict of interest.

## Footnotes

1 Personal communication with Maria Dolores de Hevia.

2 We thank a reviewer for pointing this out to us.
